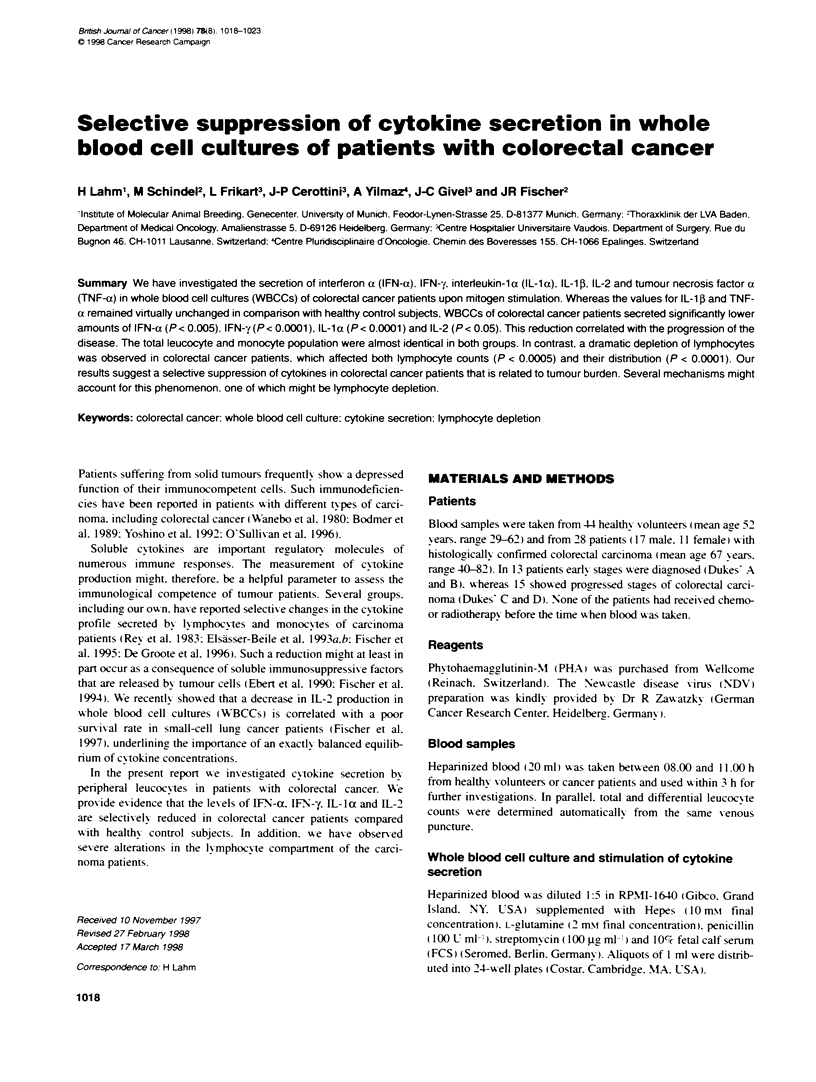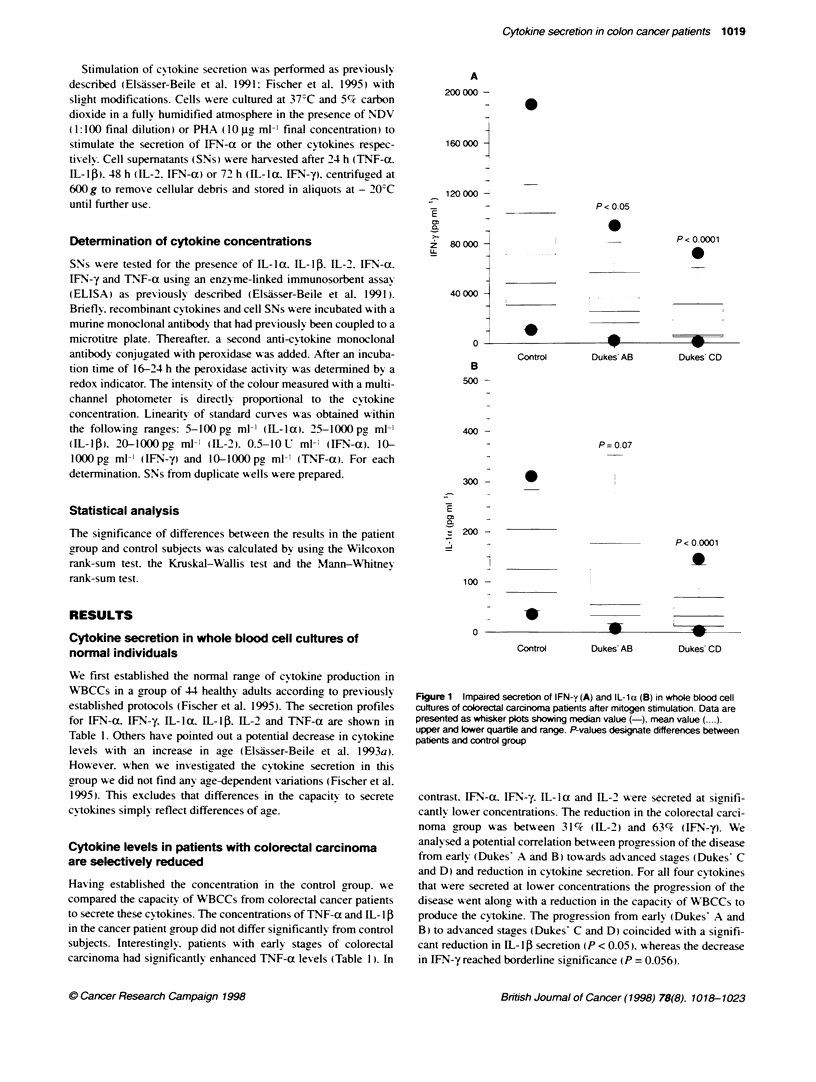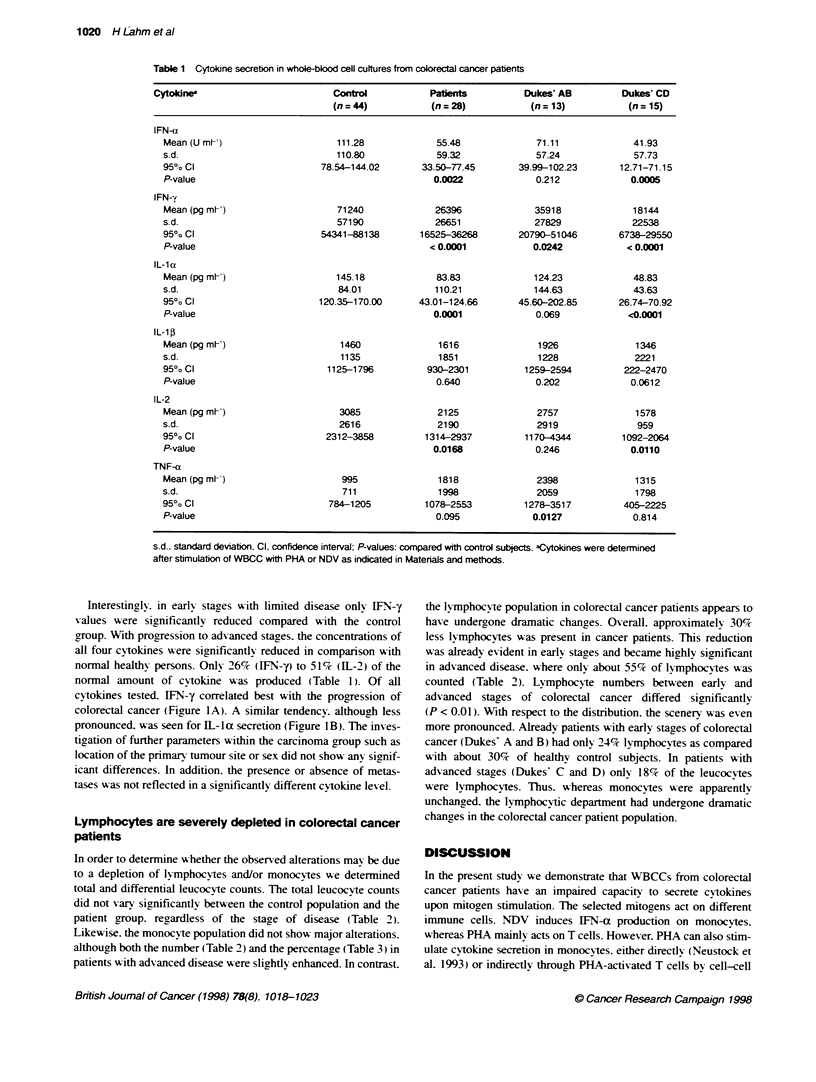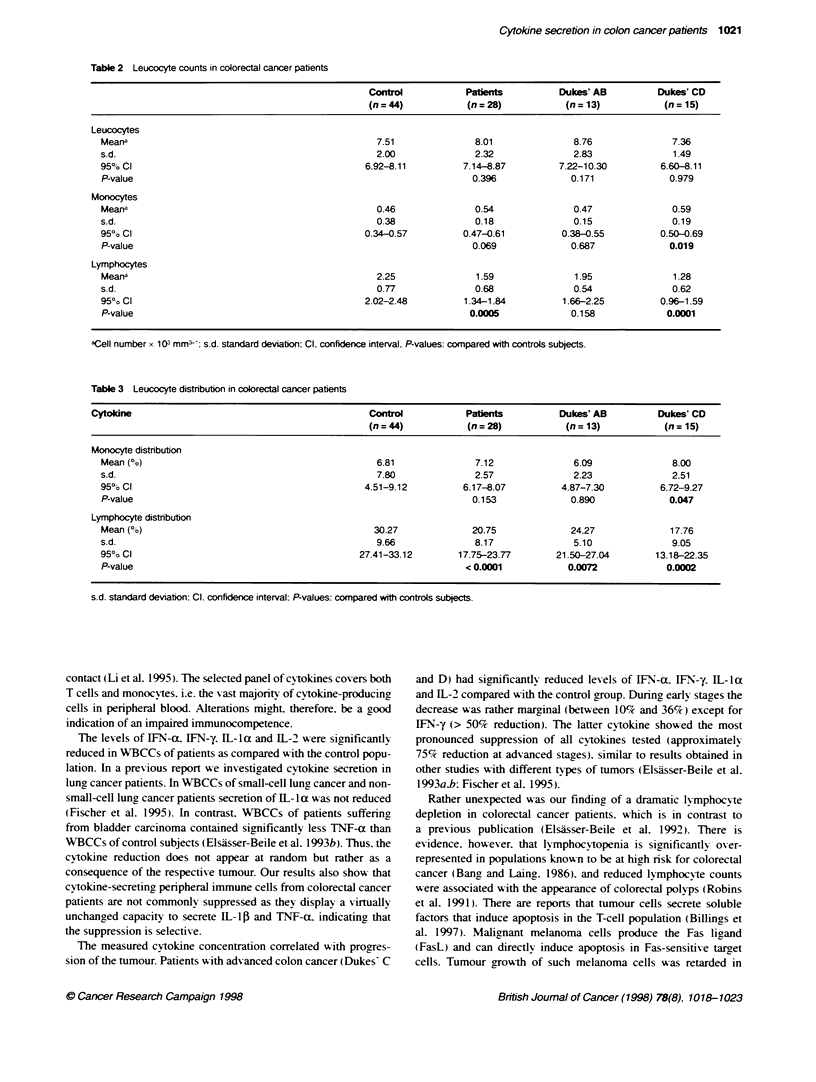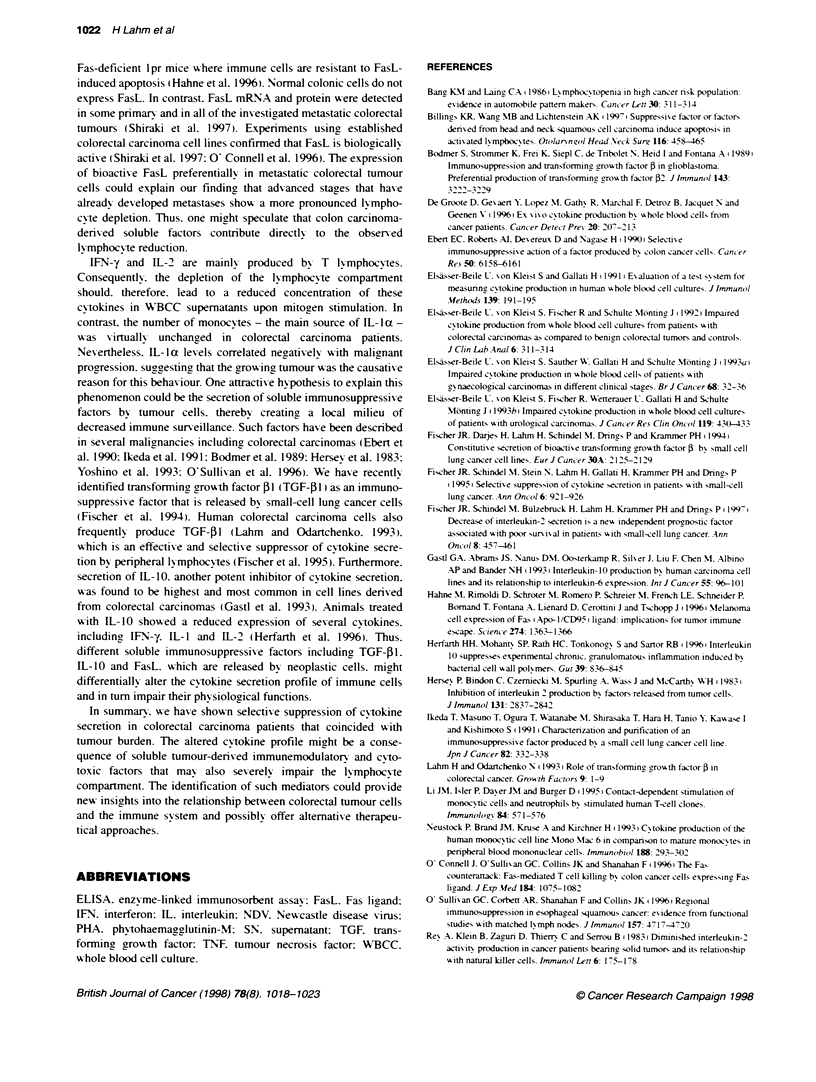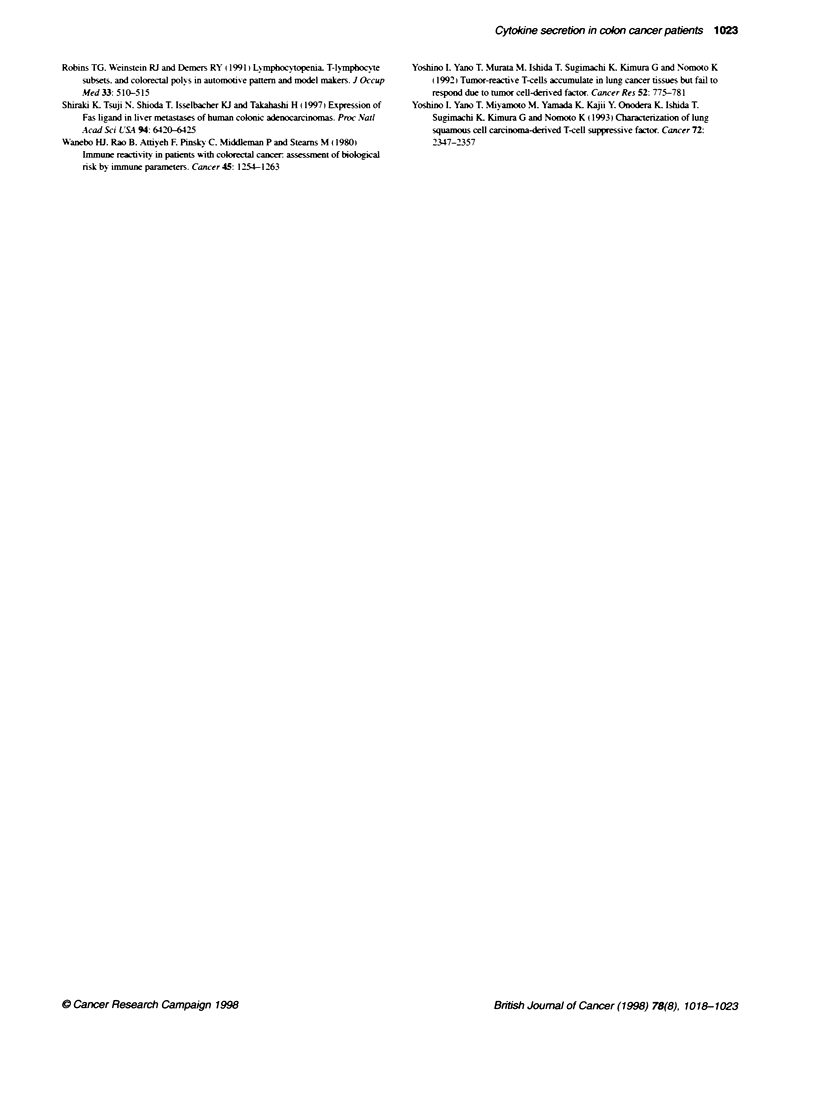# Selective suppression of cytokine secretion in whole blood cell cultures of patients with colorectal cancer.

**DOI:** 10.1038/bjc.1998.621

**Published:** 1998-10

**Authors:** H. Lahm, M. Schindel, L. Frikart, J. P. Cerottini, A. Yilmaz, J. C. Givel, J. R. Fischer

**Affiliations:** Institute of Molecular Animal Breeding, Genecenter, University of Munich, Germany.

## Abstract

We have investigated the secretion of interferon alpha (IFN-alpha), IFN-gamma, interleukin-1alpha (IL-1alpha), IL-1beta, IL-2 and tumour necrosis factor alpha (TNF-alpha) in whole blood cell cultures (WBCCs) of colorectal cancer patients upon mitogen stimulation. Whereas the values for IL-1beta and TNF-alpha remained virtually unchanged in comparison with healthy control subjects, WBCCs of colorectal cancer patients secreted significantly lower amounts of IFN-alpha (P < 0.005), IFN-gamma (P < 0.0001), IL-1alpha (P < 0.0001) and IL-2 (P < 0.05). This reduction correlated with the progression of the disease. The total leucocyte and monocyte population were almost identical in both groups. In contrast, a dramatic depletion of lymphocytes was observed in colorectal cancer patients, which affected both lymphocyte counts (P < 0.0005) and their distribution (P < 0.0001). Our results suggest a selective suppression of cytokines in colorectal cancer patients that is related to tumour burden. Several mechanisms might account for this phenomenon, one of which might be lymphocyte depletion.


British Journal of Cancer (1998) 78(8). 1018–1023
© 1998 Cancer Research Campaign

### Selective suppression of cytokine secretion in whole blood cell cultures of patients with colorectal cancer

**H Lahm[1], M Schindel[2], L Frikart[3], J-P Cerottini[3], A Yilmaz[4], J-C Givel[3] and JR Fischer[2]**

[1]Institute of Molecular Animal Breeding. Genecenter. University of Munich. Feodor-Lynen-Strasse 25. D-81377 Munich. Germany: [2]Thoraxklinik der LVA Baden. Department of Medical Oncology. Amalienstrasse 5. D-69126 Heidelberg. Germany: [3]Centre Hospitalier Universitaire Vaudois. Department of Surgery. Rue du Bugnon 46. CH-1011 Lausanne. Switzerland: [4]Centre Pluridisciplinaire d'Oncologie. Chemin des Boveresses 155. CH-1066 Epalinges. Switzerland

**Summary** We have investigated the secretion of interferon α (IFN-α). IFN-γ, interleukin-1α (IL-1α). IL-1β. IL-2 and tumour necrosis factor α (TNF-α) in whole blood cell cultures (WBCCs) of colorectal cancer patients upon mitogen stimulation. Whereas the values for IL-1β and TNF-α remained virtually unchanged in comparison with healthy control subjects. WBCCs of colorectal cancer patients secreted significantly lower amounts of IFN-α ($P < 0.005$), IFN-γ ($P < 0.0001$), IL-1α ($P < 0.0001$) and IL-2 ($P < 0.05$). This reduction correlated with the progression of the disease. The total leucocyte and monocyte population were almost identical in both groups. In contrast, a dramatic depletion of lymphocytes was observed in colorectal cancer patients, which affected both lymphocyte counts ($P < 0.0005$) and their distribution ($P < 0.0001$). Our results suggest a selective suppression of cytokines in colorectal cancer patients that is related to tumour burden. Several mechanisms might account for this phenomenon, one of which might be lymphocyte depletion.

**Keywords:** colorectal cancer: whole blood cell culture: cytokine secretion: lymphocyte depletion

Patients suffering from solid tumours frequently show a depressed function of their immunocompetent cells. Such immunodeficiencies have been reported in patients with different types of carcinoma, including colorectal cancer (Wanebo et al. 1980: Bodmer et al. 1989: Yoshino et al. 1992: O'Sullivan et al. 1996).

Soluble cytokines are important regulatory molecules of numerous immune responses. The measurement of cytokine production might, therefore, be a helpful parameter to assess the immunological competence of tumour patients. Several groups, including our own, have reported selective changes in the cytokine profile secreted by lymphocytes and monocytes of carcinoma patients (Rey et al. 1983: Elsässer-Beile et al. 1993*a,b*: Fischer et al. 1995: De Groote et al. 1996). Such a reduction might at least in part occur as a consequence of soluble immunosuppressive factors that are released by tumour cells (Ebert et al. 1990: Fischer et al. 1994). We recently showed that a decrease in IL-2 production in whole blood cell cultures (WBCCs) is correlated with a poor survival rate in small-cell lung cancer patients (Fischer et al. 1997), underlining the importance of an exactly balanced equilibrium of cytokine concentrations.

In the present report we investigated cytokine secretion by peripheral leucocytes in patients with colorectal cancer. We provide evidence that the levels of IFN-α, IFN-γ, IL-1α and IL-2 are selectively reduced in colorectal cancer patients compared with healthy control subjects. In addition, we have observed severe alterations in the lymphocyte compartment of the carcinoma patients.

*Received 10 November 1997*
*Revised 27 February 1998*
*Accepted 17 March 1998*

*Correspondence to:* H Lahm

### MATERIALS AND METHODS

### Patients

Blood samples were taken from 44 healthy volunteers (mean age 52 years, range 29–62) and from 28 patients (17 male, 11 female) with histologically confirmed colorectal carcinoma (mean age 67 years, range 40–82). In 13 patients early stages were diagnosed (Dukes' A and B), whereas 15 showed progressed stages of colorectal carcinoma (Dukes' C and D). None of the patients had received chemo- or radiotherapy before the time when blood was taken.

### Reagents

Phytohaemagglutinin-M (PHA) was purchased from Wellcome (Reinach, Switzerland). The Newcastle disease virus (NDV) preparation was kindly provided by Dr R Zawatzky (German Cancer Research Center, Heidelberg, Germany).

### Blood samples

Heparinized blood (20 ml) was taken between 08.00 and 11.00 h from healthy volunteers or cancer patients and used within 3 h for further investigations. In parallel, total and differential leucocyte counts were determined automatically from the same venous puncture.

### Whole blood cell culture and stimulation of cytokine secretion

Heparinized blood was diluted 1:5 in RPMI-1640 (Gibco, Grand Island, NY, USA) supplemented with Hepes (10 mM final concentration), L-glutamine (2 mM final concentration), penicillin (100 U $ml^{-1}$), streptomycin (100 μg $ml^{-1}$) and 10% fetal calf serum (FCS) (Seromed, Berlin, Germany). Aliquots of 1 ml were distributed into 24-well plates (Costar, Cambridge, MA, USA).

Stimulation of cytokine secretion was performed as previously described (Elsässer-Beile et al. 1991; Fischer et al. 1995) with slight modifications. Cells were cultured at 37°C and 5% carbon dioxide in a fully humidified atmosphere in the presence of NDV (1:100 final dilution) or PHA (10 µg ml$^{-1}$ final concentration) to stimulate the secretion of IFN-α or the other cytokines respectively. Cell supernatants (SNs) were harvested after 24 h (TNF-α, IL-1β), 48 h (IL-2, IFN-α) or 72 h (IL-1α, IFN-γ), centrifuged at 600 *g* to remove cellular debris and stored in aliquots at – 20°C until further use.

### Determination of cytokine concentrations

SNs were tested for the presence of IL-1α, IL-1β, IL-2, IFN-α, IFN-γ and TNF-α using an enzyme-linked immunosorbent assay (ELISA) as previously described (Elsässer-Beile et al. 1991). Briefly, recombinant cytokines and cell SNs were incubated with a murine monoclonal antibody that had previously been coupled to a microtitre plate. Thereafter, a second anti-cytokine monoclonal antibody conjugated with peroxidase was added. After an incubation time of 16–24 h the peroxidase activity was determined by a redox indicator. The intensity of the colour measured with a multichannel photometer is directly proportional to the cytokine concentration. Linearity of standard curves was obtained within the following ranges: 5–100 pg ml$^{-1}$ (IL-1α), 25–1000 pg ml$^{-1}$ (IL-1β), 20–1000 pg ml$^{-1}$ (IL-2), 0.5–10 U ml$^{-1}$ (IFN-α), 10–1000 pg ml$^{-1}$ (IFN-γ) and 10–1000 pg ml$^{-1}$ (TNF-α). For each determination, SNs from duplicate wells were prepared.

### Statistical analysis

The significance of differences between the results in the patient group and control subjects was calculated by using the Wilcoxon rank-sum test, the Kruskal–Wallis test and the Mann–Whitney rank-sum test.

### RESULTS

### Cytokine secretion in whole blood cell cultures of normal individuals

We first established the normal range of cytokine production in WBCCs in a group of 44 healthy adults according to previously established protocols (Fischer et al. 1995). The secretion profiles for IFN-α, IFN-γ, IL-1α, IL-1β, IL-2 and TNF-α are shown in Table 1. Others have pointed out a potential decrease in cytokine levels with an increase in age (Elsässer-Beile et al. 1993*a*). However, when we investigated the cytokine secretion in this group we did not find any age-dependent variations (Fischer et al. 1995). This excludes that differences in the capacity to secrete cytokines simply reflect differences of age.

### Cytokine levels in patients with colorectal carcinoma are selectively reduced

Having established the concentration in the control group, we compared the capacity of WBCCs from colorectal cancer patients to secrete these cytokines. The concentrations of TNF-α and IL-1β in the cancer patient group did not differ significantly from control subjects. Interestingly, patients with early stages of colorectal carcinoma had significantly enhanced TNF-α levels (Table 1). In contrast, IFN-α, IFN-γ, IL-1α and IL-2 were secreted at significantly lower concentrations. The reduction in the colorectal carcinoma group was between 31% (IL-2) and 63% (IFN-γ). We analysed a potential correlation between progression of the disease from early (Dukes' A and B) towards advanced stages (Dukes' C and D) and reduction in cytokine secretion. For all four cytokines that were secreted at lower concentrations the progression of the disease went along with a reduction in the capacity of WBCCs to produce the cytokine. The progression from early (Dukes' A and B) to advanced stages (Dukes' C and D) coincided with a significant reduction in IL-1β secretion ($P < 0.05$), whereas the decrease in IFN-γ reached borderline significance ($P = 0.056$).

**Figure 1** Impaired secretion of IFN-γ (**A**) and IL-1α (**B**) in whole blood cell cultures of colorectal carcinoma patients after mitogen stimulation. Data are presented as whisker plots showing median value (—), mean value (.....), upper and lower quartile and range. *P*-values designate differences between patients and control group

© Cancer Research Campaign 1998

Table 1 Cytokine secretion in whole-blood cell cultures from colorectal cancer patients

| Cytokine[a] | Control (n = 44) | Patients (n = 28) | Dukes' AB (n = 13) | Dukes' CD (n = 15) |
|---|---|---|---|---|
| IFN-α | | | | |
| Mean (U ml⁻¹) | 111.28 | 55.48 | 71.11 | 41.93 |
| s.d. | 110.80 | 59.32 | 57.24 | 57.73 |
| 95% CI | 78.54–144.02 | 33.50–77.45 | 39.99–102.23 | 12.71–71.15 |
| P-value | | **0.0022** | 0.212 | **0.0005** |
| IFN-γ | | | | |
| Mean (pg ml⁻¹) | 71240 | 26396 | 35918 | 18144 |
| s.d. | 57190 | 26651 | 27829 | 22538 |
| 95% CI | 54341–88138 | 16525–36268 | 20790–51046 | 6738–29550 |
| P-value | | **< 0.0001** | **0.0242** | **< 0.0001** |
| IL-1α | | | | |
| Mean (pg ml⁻¹) | 145.18 | 83.83 | 124.23 | 48.83 |
| s.d. | 84.01 | 110.21 | 144.63 | 43.63 |
| 95% CI | 120.35–170.00 | 43.01–124.66 | 45.60–202.85 | 26.74–70.92 |
| P-value | | **0.0001** | 0.069 | **<0.0001** |
| IL-1β | | | | |
| Mean (pg ml⁻¹) | 1460 | 1616 | 1926 | 1346 |
| s.d. | 1135 | 1851 | 1228 | 2221 |
| 95% CI | 1125–1796 | 930–2301 | 1259–2594 | 222–2470 |
| P-value | | 0.640 | 0.202 | 0.0612 |
| IL-2 | | | | |
| Mean (pg ml⁻¹) | 3085 | 2125 | 2757 | 1578 |
| s.d. | 2616 | 2190 | 2919 | 959 |
| 95% CI | 2312–3858 | 1314–2937 | 1170–4344 | 1092–2064 |
| P-value | | **0.0168** | 0.246 | **0.0110** |
| TNF-α | | | | |
| Mean (pg ml⁻¹) | 995 | 1818 | 2398 | 1315 |
| s.d. | 711 | 1998 | 2059 | 1798 |
| 95% CI | 784–1205 | 1078–2553 | 1278–3517 | 405–2225 |
| P-value | | 0.095 | **0.0127** | 0.814 |

s.d., standard deviation, CI, confidence interval; P-values: compared with control subjects. [a]Cytokines were determined after stimulation of WBCC with PHA or NDV as indicated in Materials and methods.

Interestingly, in early stages with limited disease only IFN-γ values were significantly reduced compared with the control group. With progression to advanced stages, the concentrations of all four cytokines were significantly reduced in comparison with normal healthy persons. Only 26% (IFN-γ) to 51% (IL-2) of the normal amount of cytokine was produced (Table 1). Of all cytokines tested, IFN-γ correlated best with the progression of colorectal cancer (Figure 1A). A similar tendency, although less pronounced, was seen for IL-1α secretion (Figure 1B). The investigation of further parameters within the carcinoma group such as location of the primary tumour site or sex did not show any significant differences. In addition, the presence or absence of metastases was not reflected in a significantly different cytokine level.

### Lymphocytes are severely depleted in colorectal cancer patients

In order to determine whether the observed alterations may be due to a depletion of lymphocytes and/or monocytes we determined total and differential leucocyte counts. The total leucocyte counts did not vary significantly between the control population and the patient group, regardless of the stage of disease (Table 2). Likewise, the monocyte population did not show major alterations, although both the number (Table 2) and the percentage (Table 3) in patients with advanced disease were slightly enhanced. In contrast, the lymphocyte population in colorectal cancer patients appears to have undergone dramatic changes. Overall, approximately 30% less lymphocytes was present in cancer patients. This reduction was already evident in early stages and became highly significant in advanced disease, where only about 55% of lymphocytes was counted (Table 2). Lymphocyte numbers between early and advanced stages of colorectal cancer differed significantly ($P < 0.01$). With respect to the distribution, the scenery was even more pronounced. Already patients with early stages of colorectal cancer (Dukes' A and B) had only 24% lymphocytes as compared with about 30% of healthy control subjects. In patients with advanced stages (Dukes' C and D) only 18% of the leucocytes were lymphocytes. Thus, whereas monocytes were apparently unchanged, the lymphocytic department had undergone dramatic changes in the colorectal cancer patient population.

### DISCUSSION

In the present study we demonstrate that WBCCs from colorectal cancer patients have an impaired capacity to secrete cytokines upon mitogen stimulation. The selected mitogens act on different immune cells. NDV induces IFN-α production on monocytes, whereas PHA mainly acts on T cells. However, PHA can also stimulate cytokine secretion in monocytes, either directly (Neustock et al, 1993) or indirectly through PHA-activated T cells by cell–cell

© Cancer Research Campaign 1998

**Table 2** Leucocyte counts in colorectal cancer patients

| | Control ($n$ = 44) | Patients ($n$ = 28) | Dukes' AB ($n$ = 13) | Dukes' CD ($n$ = 15) |
|---|---|---|---|---|
| Leucocytes | | | | |
| Mean[a] | 7.51 | 8.01 | 8.76 | 7.36 |
| s.d. | 2.00 | 2.32 | 2.83 | 1.49 |
| 95% CI | 6.92–8.11 | 7.14–8.87 | 7.22–10.30 | 6.60–8.11 |
| $P$-value | | 0.396 | 0.171 | 0.979 |
| Monocytes | | | | |
| Mean[a] | 0.46 | 0.54 | 0.47 | 0.59 |
| s.d. | 0.38 | 0.18 | 0.15 | 0.19 |
| 95% CI | 0.34–0.57 | 0.47–0.61 | 0.38–0.55 | 0.50–0.69 |
| $P$-value | | 0.069 | 0.687 | **0.019** |
| Lymphocytes | | | | |
| Mean[a] | 2.25 | 1.59 | 1.95 | 1.28 |
| s.d. | 0.77 | 0.68 | 0.54 | 0.62 |
| 95% CI | 2.02–2.48 | 1.34–1.84 | 1.66–2.25 | 0.96–1.59 |
| $P$-value | | **0.0005** | 0.158 | **0.0001** |

[a]Cell number × $10^3$ mm$^{3-1}$; s.d. standard deviation; CI, confidence interval. $P$-values: compared with controls subjects.

**Table 3** Leucocyte distribution in colorectal cancer patients

| Cytokine | Control ($n$ = 44) | Patients ($n$ = 28) | Dukes' AB ($n$ = 13) | Dukes' CD ($n$ = 15) |
|---|---|---|---|---|
| Monocyte distribution | | | | |
| Mean (%) | 6.81 | 7.12 | 6.09 | 8.00 |
| s.d. | 7.80 | 2.57 | 2.23 | 2.51 |
| 95% CI | 4.51-9.12 | 6.17–8.07 | 4.87–7.30 | 6.72–9.27 |
| $P$-value | | 0.153 | 0.890 | **0.047** |
| Lymphocyte distribution | | | | |
| Mean (%) | 30.27 | 20.75 | 24.27 | 17.76 |
| s.d. | 9.66 | 8.17 | 5.10 | 9.05 |
| 95% CI | 27.41–33.12 | 17.75–23.77 | 21.50–27.04 | 13.18–22.35 |
| $P$-value | | **< 0.0001** | **0.0072** | **0.0002** |

s.d. standard deviation; CI, confidence interval; $P$-values: compared with controls subjects.

contact (Li et al, 1995). The selected panel of cytokines covers both T cells and monocytes, i.e. the vast majority of cytokine-producing cells in peripheral blood. Alterations might, therefore, be a good indication of an impaired immunocompetence.

The levels of IFN-α, IFN-γ, IL-1α and IL-2 were significantly reduced in WBCCs of patients as compared with the control population. In a previous report we investigated cytokine secretion in lung cancer patients. In WBCCs of small-cell lung cancer and non-small-cell lung cancer patients secretion of IL-1α was not reduced (Fischer et al, 1995). In contrast, WBCCs of patients suffering from bladder carcinoma contained significantly less TNF-α than WBCCs of control subjects (Elsässer-Beile et al, 1993*b*). Thus, the cytokine reduction does not appear at random but rather as a consequence of the respective tumour. Our results also show that cytokine-secreting peripheral immune cells from colorectal cancer patients are not commonly suppressed as they display a virtually unchanged capacity to secrete IL-1β and TNF-α, indicating that the suppression is selective.

The measured cytokine concentration correlated with progression of the tumour. Patients with advanced colon cancer (Dukes' C and D) had significantly reduced levels of IFN-α, IFN-γ, IL-1α and IL-2 compared with the control group. During early stages the decrease was rather marginal (between 10% and 36%) except for IFN-γ (> 50% reduction). The latter cytokine showed the most pronounced suppression of all cytokines tested (approximately 75% reduction at advanced stages), similar to results obtained in other studies with different types of tumors (Elsässer-Beile et al, 1993*a*,*b*; Fischer et al, 1995).

Rather unexpected was our finding of a dramatic lymphocyte depletion in colorectal cancer patients, which is in contrast to a previous publication (Elsässer-Beile et al, 1992). There is evidence, however, that lymphocytopenia is significantly over-represented in populations known to be at high risk for colorectal cancer (Bang and Laing, 1986), and reduced lymphocyte counts were associated with the appearance of colorectal polyps (Robins et al, 1991). There are reports that tumour cells secrete soluble factors that induce apoptosis in the T-cell population (Billings et al, 1997). Malignant melanoma cells produce the Fas ligand (FasL) and can directly induce apoptosis in Fas-sensitive target cells. Tumour growth of such melanoma cells was retarded in

© Cancer Research Campaign 1998

Fas-deficient lpr mice where immune cells are resistant to FasL-induced apoptosis (Hahne et al. 1996). Normal colonic cells do not express FasL. In contrast, FasL mRNA and protein were detected in some primary and in all of the investigated metastatic colorectal tumours (Shiraki et al. 1997). Experiments using established colorectal carcinoma cell lines confirmed that FasL is biologically active (Shiraki et al. 1997; O' Connell et al. 1996). The expression of bioactive FasL preferentially in metastatic colorectal tumour cells could explain our finding that advanced stages that have already developed metastases show a more pronounced lymphocyte depletion. Thus, one might speculate that colon carcinoma-derived soluble factors contribute directly to the observed lymphocyte reduction.

IFN-γ and IL-2 are mainly produced by T lymphocytes. Consequently, the depletion of the lymphocyte compartment should, therefore, lead to a reduced concentration of these cytokines in WBCC supernatants upon mitogen stimulation. In contrast, the number of monocytes – the main source of IL-1α – was virtually unchanged in colorectal carcinoma patients. Nevertheless, IL-1α levels correlated negatively with malignant progression, suggesting that the growing tumour was the causative reason for this behaviour. One attractive hypothesis to explain this phenomenon could be the secretion of soluble immunosuppressive factors by tumour cells, thereby creating a local milieu of decreased immune surveillance. Such factors have been described in several malignancies including colorectal carcinomas (Ebert et al. 1990; Ikeda et al. 1991; Bodmer et al. 1989; Hersey et al. 1983; Yoshino et al. 1993; O'Sullivan et al. 1996). We have recently identified transforming growth factor β1 (TGF-β1) as an immunosuppressive factor that is released by small-cell lung cancer cells (Fischer et al. 1994). Human colorectal carcinoma cells also frequently produce TGF-β1 (Lahm and Odartchenko, 1993), which is an effective and selective suppressor of cytokine secretion by peripheral lymphocytes (Fischer et al. 1995). Furthermore, secretion of IL-10, another potent inhibitor of cytokine secretion, was found to be highest and most common in cell lines derived from colorectal carcinomas (Gastl et al. 1993). Animals treated with IL-10 showed a reduced expression of several cytokines, including IFN-γ, IL-1 and IL-2 (Herfarth et al. 1996). Thus, different soluble immunosuppressive factors including TGF-β1, IL-10 and FasL, which are released by neoplastic cells, might differentially alter the cytokine secretion profile of immune cells and in turn impair their physiological functions.

In summary, we have shown selective suppression of cytokine secretion in colorectal carcinoma patients that coincided with tumour burden. The altered cytokine profile might be a consequence of soluble tumour-derived immunemodulatory and cytotoxic factors that may also severely impair the lymphocyte compartment. The identification of such mediators could provide new insights into the relationship between colorectal tumour cells and the immune system and possibly offer alternative therapeutical approaches.

### ABBREVIATIONS

ELISA, enzyme-linked immunosorbent assay; FasL, Fas ligand; IFN, interferon; IL, interleukin; NDV, Newcastle disease virus; PHA, phytohaemagglutinin-M; SN, supernatant; TGF, transforming growth factor; TNF, tumour necrosis factor; WBCC, whole blood cell culture.

### REFERENCES

Bang KM and Laing CA (1986) Lymphocytopenia in high cancer risk population: evidence in automobile pattern makers. *Cancer Lett* **30**: 311–314

Billings KR, Wang MB and Lichtenstein AK (1997) Suppressive factor or factors derived from head and neck squamous cell carcinoma induce apoptosis in activated lymphocytes. *Otolaryngol Head Neck Surg* **116**: 458–465

Bodmer S, Strommer K, Frei K, Siepl C, de Tribolet N, Heid I and Fontana A (1989) Immunosuppression and transforming growth factor β in glioblastoma. Preferential production of transforming growth factor β2. *J Immunol* **143**: 3222–3229

De Groote D, Gevaert Y, Lopez M, Gathy R, Marchal F, Detroz B, Jacquet N and Geenen V (1996) Ex vivo cytokine production by whole blood cells from cancer patients. *Cancer Detect Prev* **20**: 207–213

Ebert EC, Roberts AI, Devereux D and Nagase H (1990) Selective immunosuppressive action of a factor produced by colon cancer cells. *Cancer Res* **50**: 6158–6161

Elsässer-Beile U, von Kleist S and Gallati H (1991) Evaluation of a test system for measuring cytokine production in human whole blood cell cultures. *J Immunol Methods* **139**: 191–195

Elsässer-Beile U, von Kleist S, Fischer R and Schulte Mönting J (1992) Impaired cytokine production from whole blood cell cultures from patients with colorectal carcinomas as compared to benign colorectal tumors and controls. *J Clin Lab Anal* **6**: 311–314

Elsässer-Beile U, von Kleist S, Sauther W, Gallati H and Schulte Mönting J (1993*a*) Impaired cytokine production in whole blood cells of patients with gynaecological carcinomas in different clinical stages. *Br J Cancer* **68**: 32–36

Elsässer-Beile U, von Kleist S, Fischer R, Wetterauer U, Gallati H and Schulte Mönting J (1993*b*) Impaired cytokine production in whole blood cell cultures of patients with urological carcinomas. *J Cancer Res Clin Oncol* **119**: 430–433

Fischer JR, Darjes H, Lahm H, Schindel M, Drings P and Krammer PH (1994) Constitutive secretion of bioactive transforming growth factor β, by small cell lung cancer cell lines. *Eur J Cancer* **30A**: 2125–2129

Fischer JR, Schindel M, Stein N, Lahm H, Gallati H, Krammer PH and Drings P (1995) Selective suppression of cytokine secretion in patients with small-cell lung cancer. *Ann Oncol* **6**: 921–926

Fischer JR, Schindel M, Bülzebruck H, Lahm H, Krammer PH and Drings P (1997) Decrease of interleukin-2 secretion is a new independent prognostic factor associated with poor survival in patients with small-cell lung cancer. *Ann Oncol* **8**: 457–461

Gastl GA, Abrams JS, Nanus DM, Oosterkamp R, Silver J, Liu F, Chen M, Albino AP and Bander NH (1993) Interleukin-10 production by human carcinoma cell lines and its relationship to interleukin-6 expression. *Int J Cancer* **55**: 96–101

Hahne M, Rimoldi D, Schroter M, Romero P, Schreier M, French LE, Schneider P, Bornand T, Fontana A, Lienard D, Cerottini J and Tschopp J (1996) Melanoma cell expression of Fas (Apo-1/CD95) ligand: implications for tumor immune escape. *Science* **274**: 1363–1366

Herfarth HH, Mohanty SP, Rath HC, Tonkonogy S and Sartor RB (1996) Interleukin 10 suppresses experimental chronic, granulomatous inflammation induced by bacterial cell wall polymers. *Gut* **39**: 836–845

Hersey P, Bindon C, Czerniecki M, Spurling A, Wass J and McCarthy WH (1983) Inhibition of interleukin 2 production by factors released from tumor cells. *J Immunol* **131**: 2837–2842

Ikeda T, Masuno T, Ogura T, Watanabe M, Shirasaka T, Hara H, Tanio Y, Kawase I and Kishimoto S (1991) Characterization and purification of an immunosuppressive factor produced by a small cell lung cancer cell line. *Jpn J Cancer* **82**: 332–338

Lahm H and Odartchenko N (1993) Role of transforming growth factor β in colorectal cancer. *Growth Factors* **9**: 1–9

Li JM, Isler P, Dayer JM and Burger D (1995) Contact-dependent stimulation of monocytic cells and neutrophils by stimulated human T-cell clones. *Immunology* **84**: 571–576

Neustock P, Brand JM, Kruse A and Kirchner H (1993) Cytokine production of the human monocytic cell line Mono Mac 6 in comparison to mature monocytes in peripheral blood mononuclear cells. *Immunobiol* **188**: 293–302

O' Connell J, O'Sullivan GC, Collins JK and Shanahan F (1996) The Fas counterattack: Fas-mediated T cell killing by colon cancer cells expressing Fas ligand. *J Exp Med* **184**: 1075–1082

O' Sullivan GC, Corbett AR, Shanahan F and Collins JK (1996) Regional immunosuppression in esophageal squamous cancer: evidence from functional studies with matched lymph nodes. *J Immunol* **157**: 4717–4720

Rey A, Klein B, Zaguri D, Thierry C and Serrou B (1983) Diminished interleukin-2 activity production in cancer patients bearing solid tumors and its relationship with natural killer cells. *Immunol Lett* **6**: 175–178

© Cancer Research Campaign 1998

Robins TG. Weinstein RJ and Demers RY (1991) Lymphocytopenia. T-lymphocyte subsets. and colorectal polys in automotive pattern and model makers. *J Occup Med* **33**: 510–515

Shiraki K. Tsuji N. Shioda T. Isselbacher KJ and Takahashi H (1997) Expression of Fas ligand in liver metastases of human colonic adenocarcinomas. *Proc Natl Acad Sci USA* **94**: 6420–6425

Wanebo HJ. Rao B. Attiyeh F. Pinsky C. Middleman P and Stearns M (1980) Immune reactivity in patients with colorectal cancer: assessment of biological risk by immune parameters. *Cancer* **45**: 1254–1263

Yoshino I. Yano T. Murata M. Ishida T. Sugimachi K. Kimura G and Nomoto K (1992) Tumor-reactive T-cells accumulate in lung cancer tissues but fail to respond due to tumor cell-derived factor. *Cancer Res* **52**: 775–781

Yoshino I. Yano T. Miyamoto M. Yamada K. Kajii Y. Onodera K. Ishida T. Sugimachi K. Kimura G and Nomoto K (1993) Characterization of lung squamous cell carcinoma-derived T-cell suppressive factor. *Cancer* **72**: 2347–2357

© Cancer Research Campaign 1998